# Risk of stillbirth and neonatal death in singletons born after fresh and frozen embryo transfer. Cohort study from the Committee of Nordic Assisted Reproduction Technology and Safety

**DOI:** 10.1016/j.fertnstert.2022.10.020

**Published:** 2022-12-24

**Authors:** Kjersti Westvik-Johari, Deborah A Lawlor, Liv Bente Romundstad, Christina Bergh, Ulla-Britt Wennerholm, Mika Gissler, Anna-Karina A Henningsen, Siri E Håberg, Aila Tiitinen, Anne Lærke Spangmose, Anja Pinborg, Signe Opdahl

**Affiliations:** 1 Department of Fertility, Women and Children’s’ Centre, St Olavs Hospital, Trondheim, Norway; 2 Department of Public Health and Nursing, Norwegian University of Science and Technology, Trondheim, Post box 8905, 7491 Trondheim, Norway; 3 MRC Integrative Epidemiology Unit at the University of Bristol, Bristol, UK; 4 Population Health Science, Bristol Medical School, Bristol, UK; 5 NIHR Bristol Biomedical Research Centre, Bristol, UK; 6 Centre for Fertility and Health, Norwegian Institute of Public Health, Oslo, Norway; 7 Spiren Fertility Clinic, Trondheim, Norway; 8 Department of Obstetrics and Gynaecology, Institute of Clinical Sciences, Sahlgrenska Academy, University of Gothenburg, Sahlgrenska University Hospital, Gothenburg, Sweden; 9 THL Finnish Institute for Health and Welfare, Department of Knowledge Brokers, Helsinki, Finland; 10 Karolinska Institutet, Department of Molecular Medicine and Surgery, Stockholm, Sweden and Region Stockholm, Academic Primary Health Care Centre, Stockholm, Sweden; 11 The Fertility Clinic, Copenhagen University Hospital, Rigshospitalet, Copenhagen, Denmark; 12 Department of Obstetrics and Gynaecology, Helsinki University Hospital and University of Helsinki, Finland

**Keywords:** Neonatal death, stillbirth, assisted conception, IVF, ART

## Abstract

**Objectives:**

To investigate whether risks of stillbirth and neonatal death differ after fresh and frozen embryo transfer (fresh and frozen-ET) compared to singletons conceived without medical assistance.

**Design:**

Population-based cohort.

**Setting:**

Data linkage between the nationwide Medical Birth Registries in Denmark (1994-2014), Norway and Sweden (1988-2015), and national quality registries and databases on assisted reproductive technology.

**Patients:**

4,590,853 singletons, including 78,642 conceived by fresh transfer and 18,084 by frozen-ET.

**Intervention:**

None

**Main Outcome Measures:**

Stillbirth (fetal death before and during delivery) and neonatal death (live born with death 0-27 days postpartum).

**Results:**

Overall, 17,123 (0.37%) singletons were stillborn and 7,685 (0.17%) died neonatally. Compared to singletons conceived without medical assistance, the odds of stillbirth were similar after fresh and frozen-ET, while odds of neonatal death were higher after fresh (odds ratio [OR] 1.69, 95% confidence interval [CI] 1.46-1.95) and frozen-ET (OR 1.51, 95% CI 1.08-2.10).

Preterm birth (<37 gestational weeks) was more common after fresh (8.0%) and frozen-ET (6.6%) compared to singletons conceived without medical assistance (5.0%), and strongly associated with neonatal mortality across all conception methods. Within gestational age categories, risk of stillbirth and neonatal death was similar for all conception methods, except that singletons from fresh-ET had a higher risk of stillbirth during gestational week 22-27 (OR 1.85, 95% CI 1.51-2.26).

**Conclusion:**

Overall risk of stillbirth was similar after fresh and frozen-ET compared to singletons conceived without medical assistance, whereas neonatal mortality was higher, possibly mediated by the higher risk of preterm birth when compared to singletons conceived without medical assistance. Our results gave no clear support for choosing one treatment over the other.

## Introduction

From its experimental beginning, assisted reproductive technology (ART) has grown into a successful treatment with >9 million children born worldwide.(1, 2) While most children born after ART so far were conceived by fresh embryo transfer (fresh-ET), the number of children conceived by frozen embryo transfer (frozen-ET) has surged over the last decade,(3, 4) now comprising >50% of all children born after ART in many high-income countries.(5, 6)

Perinatal loss, whether stillbirth or neonatal death, is a traumatic outcome for expecting couples and often associated with preterm birth, congenital malformations, and placental complications.(7–11)Studies show higher risk of perinatal death overall(12, 13) and of stillbirth(14) after any ART compared to singletons conceived without medical assistance. Only one previous cohort study has separated perinatal mortality into stillbirth and neonatal death, and found higher risk of both stillbirth and neonatal death after any-ART vs conceptions without medical assistance.(15) Investigating risk of stillbirth and neonatal death in the same population is important, as neonatal death also reflects early neonatal care in addition to intra- and antepartum factors.(16) Moreover, guidelines for antepartum surveillance and induction of labor are often based on risk of stillbirth rather than neonatal death.(1, 17, 18)

Further, the increasing use of frozen-ET highlights the need to differentiate between different types of ART when assessing risk of stillbirth and neonatal death, which could not be done in most previous studies.(13–15, 19, 20) Two small cohort studies have indicated higher crude risk of neonatal death after frozen-ET compared to fresh-ET.(21, 22) A cohort study of births in the Nordic countries 1988-2007, matched on parity and year of birth, found that singletons conceived by frozen-ET had similar risks of stillbirth and neonatal death compared to fresh-ET, but higher risk of neonatal death compared to singletons conceived without medical assistance.(23)

Singletons conceived after both fresh-ET and frozen-ET are at increased risk of adverse perinatal outcomes, including preterm birth,(1, 17, 18) which is a major contributor to neonatal death.(8, 24) It is not known whether pregnancies after fresh-ET and pregnancies after frozen-ET are at higher risk of stillbirth or neonatal death during specific gestational ages or to what extent their higher risk of preterm birth contributes to neonatal mortality. An Australian study reported lower perinatal mortality in ART-conceived born <32 weeks compared to singletons conceived without medical assistance, despite a higher overall risk of stillbirth and neonatal death among ART-conceived.(20) Contrary, in the previously mentioned Nordic, matched cohort, a higher risk of stillbirth among singletons conceived by any ART was found before 28 weeks’ gestation, and a similar risk of stillbirth overall, but a higher risk of neonatal death.(19) These apparently conflicting results may be due to differences in analytical approaches, study populations and outcome definitions.

In this study we compared the risk of stillbirth and neonatal death in singletons born after fresh-ET and frozen-ET to singletons born without medical assistance, in a large Nordic population of births during 1988-2015. We also estimated risk according to gestational age.

## Materials and Methods

### Data sources and study variables

This study is based on the Committee of Nordic ART and Safety (CoNARTaS) cohort which includes data on all births registered in the nationwide Medical Birth Registries in Denmark (1994-2014), Norway (1984-2015) and Sweden (1985-2015).(4) ART-conceptions were identified through data linkage with the national ART registries and databases, using the unique national identity number assigned to each resident. The registration of ART-conception began at different times in each country. In Denmark, all ART cycles were registered in the national ART registry from 1994, including public and private clinics.(25) From 1984, public and private ART clinics in Norway reported to the Medical Birth Registry for all ART cycles that result in pregnancy verified by ultrasound in gestational week 6–7. In Sweden, the National Board of Health and Welfare received information on all deliveries after ART from 1982 to 2006, and since 2007, the National Quality Registry for Assisted Reproduction collects information on all ART cycles.

Exposures were fresh or frozen embryo transfer. Pregnancies with no ART registration was considered as conceptions without medical assistance. Our main outcomes were stillbirth and neonatal death, as defined by the Medical Birth Registry in each country. Until April 2004 in Denmark and July 2008 in Sweden, stillbirths were defined as deliveries at ≥28+0 weeks’ gestation with fetal death before or during delivery, thereafter the definition was expanded to include deliveries ≥22+0 weeks. Norway registered stillbirths delivered at ≥22+0 weeks throughout the study period. Live births were registered at any gestational age throughout the study period in all countries. Neonatal death was defined as a liveborn who died 0-27 days after birth.(26)

In pregnancies conceived without medical assistanece, gestational age was estimated from first (Denmark) or second (Norway and Sweden) trimester ultrasound examination when available, otherwise last menstrual period was used. For ART pregnancies, gestational age was estimated from embryo transfer date in Sweden. In Denmark and Norway, gestational age was estimated from ultrasound examination, or transfer date if ultrasound data were missing. We categorized gestational age as 22-27 (extremely preterm), 28-31 (very preterm), 32-36 (moderate to late preterm), 37-41 (term), and 42-44 (post-term).(27) We used Marsal’s equations for intrauterine growth to estimate z-scores of birthweights where one standard deviation was set to 11% of the expected birthweight according to sex and gestational age in days.(28)

Maternal height and pre-pregnancy or first trimester weight were registered from 2007 in Norway, 2004 in Denmark and throughout the study period in Sweden, apart from in 1990 and 1991. Maternal body mass index (BMI) was calculated as weight in kilograms divided by height in meters squared. Maternal smoking habits were registered from 1999 in Norway and during the entire study period in Denmark and Sweden and was categorized as any versus no smoking during pregnancy. The number of embryos transferred in ART-conceived pregnancies were recorded in all countries during the entire study period. Culture duration in days was recorded from 1994 in Denmark, 2011 in Norway and 1985 in Sweden, and categorized as day 2-3 (cleavage stage embryo) and 5-6 (blastocyst). Placental complications included placental abruption, placenta previa and hypertensive disorders in pregnancy as described previously.(29)

Causes of death were available from the Cause of Death Registry in each country for the liveborn population. Causes of neonatal death were categorized as recommended by the World Health Organization.(30)

### Study populations

**Figure 1:**
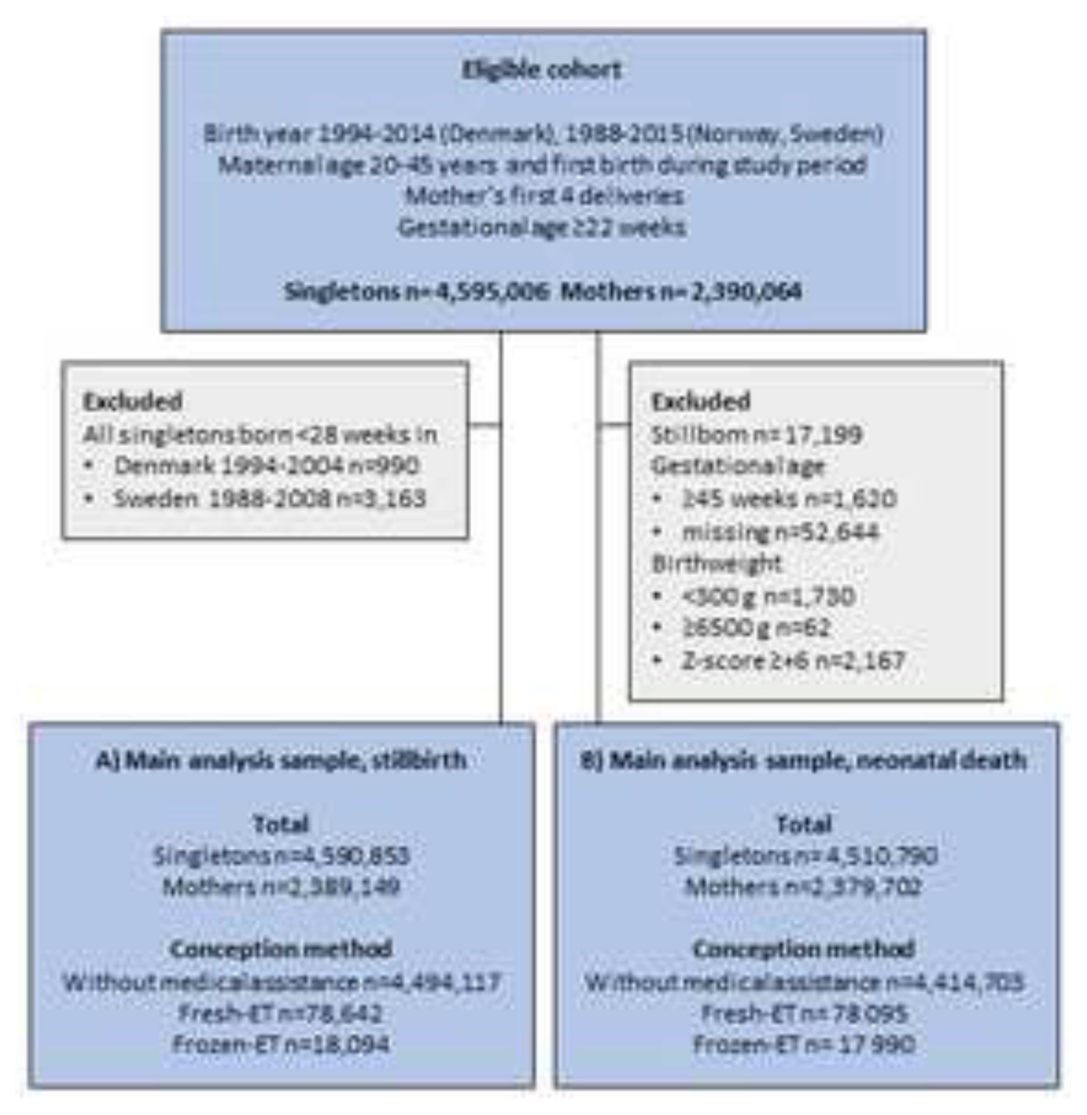
Flow chart of the study population: eligibility and exclusion criteria and numbers included in the main analysis samples. Z-score of birthweights was calculated according to gestational age (in days) and sex.

The first delivery after frozen-ET was registered in 1988. Eligibility was defined as all singleton deliveries in 1988 or later, with known conception method and parity, by women who gave their first birth during the study period (Figure 1). Additional eligibility criteria were maternal age 20-45 years and no more than the first four deliveries for each mother, as very few mothers who conceived by ART had more than four deliveries.

For analyses of stillbirth, we included stillborn and live born singletons ≥22 weeks from Norway throughout the study period, while from Denmark and Sweden we included stillborn and live born ≥28 weeks until April 2004 and July 2008 respectively, thereafter all stillborn and live born ≥22 weeks (Figure 1, sample A). No birthweight restrictions were applied in analyses of stillbirth (31). In total 4,590,853 singletons, including 78,642 after fresh-ET and 18,094 after frozen-ET were included in the main analyses for stillbirth.

To analyze neonatal death, we included only live born singletons with gestational age 22-44 weeks and excluded singletons with extreme (<300g, ≥6500g, or z-score ≥6) or missing birthweights (Figure 1, sample B). In total 4,510,790 infants were included in the main analyses for neonatal death, with 78,095 singletons conceived after fresh-ET and 17,990 after frozen-ET.

### Statistical analysis

We used random effects logistic models to compare outcomes across conception methods, with pregnancies as one level and mothers as another, estimating odds ratios (ORs) with 95% confidence intervals (CIs). To increase interpretability, we used post estimation commands to obtain risk differences. Potential confounders were factors previously shown to influence both stillbirth or neonatal death and the need for ART. Based on available information, we made the following adjustments: year of birth (continuous), country, maternal age (continuous), and parity.

We examined the consistency of results by repeating analyses in samples with 1) recorded maternal weight, height, and smoking status, where we adjusted for BMI, height, and smoking status, 2) primiparous women only, 3) ART-conceptions restricted to single embryo transfer, and 4) ART-conceptions restricted to blastocyst transfer and pregnancies conceived without medical assistance restricted to birth years when blastocyst culture was recorded. For stillbirths, we repeated the analyses in a sample restricted to deliveries ≥28 weeks to examine the potential impact of different definitions of stillbirth. To facilitate comparison with other studies, we also analyzed early neonatal deaths, defined as live born children who died 0-6 days after birth(23, 32, 33). Finally, we estimated country-specific associations.

To investigate whether conception method modified the impact of gestational age on risk of stillbirth and neonatal death, we repeated analyses within categories of gestational age. For stillbirth, we used ‘fetuses at risk’ as the denominator (i.e. all pregnancies at risk of stillbirth at the start of a given gestational age interval), with logistic models for categorical estimates and survival analysis for continuous gestational age.(34) For neonatal death, the denominator was singletons born alive during the given interval, with logistic models for categories and single weeks of gestational age. To quantify the impact of conception method on selection into the liveborn population, we repeated the main and gestational-age specific analyses using fetuses at risk as the denominator (further described in Supplemental Materials).(16)

### Ethical Considerations

In Denmark, ethical approval is not required for anonymised registry-based research. In Norway, ethical approval was given by the Regional Committee for Medical and Health Research Ethics (REK-Nor: 2010/1909-1-24, 14398). In Sweden, approval was obtained from the Ethical committee in Gothenburg (Dnr 214–12, T422-12, T516-15, T233-16, T300-17, T1144-17, and T121-18).

## Results

Table 1 describes our total study population, stillbirths and neonatal deaths. Stillbirth occurred in 17,123 pregnancies (0.37%), whereas 7,685 of all liveborn singletons died in the neonatal period (0.17%). Women who experienced stillbirth or neonatal death had higher mean BMI, were more often primiparous and less likely to have received single embryo transfer.

Compared to pregnancies conceived without medical assistance, pregnancies after fresh and frozen-ET were at higher risk of preterm birth (<37 weeks, 8.1% and 6.6% versus 5.0%). In all conception groups, preterm birth was substantially more common in cases of stillbirth and neonatal death. Malformations were also common among infants who died neonatally, with higher prevalence among singletons born without medical assistance, than after fresh-ET and frozen-ET. For stillbirths, the reported prevalence of malformations was low across all conception methods, possibly due to autopsy reports being completed after birth reports are sent to the Medical Birth Registries. Placental complications were more common after fresh and frozen-ET compared to pregnancies conceived without medical assistance. This pattern was similar but with higher incidence in pregnancies ending with stillbirth or neonatal death. The main causes of neonatal death reported to the Cause of Death Registry were malformations, maternal conditions and preterm birth, where the latter two were more common after ART conception (Supplemental Table 1).

**Figure 2:**
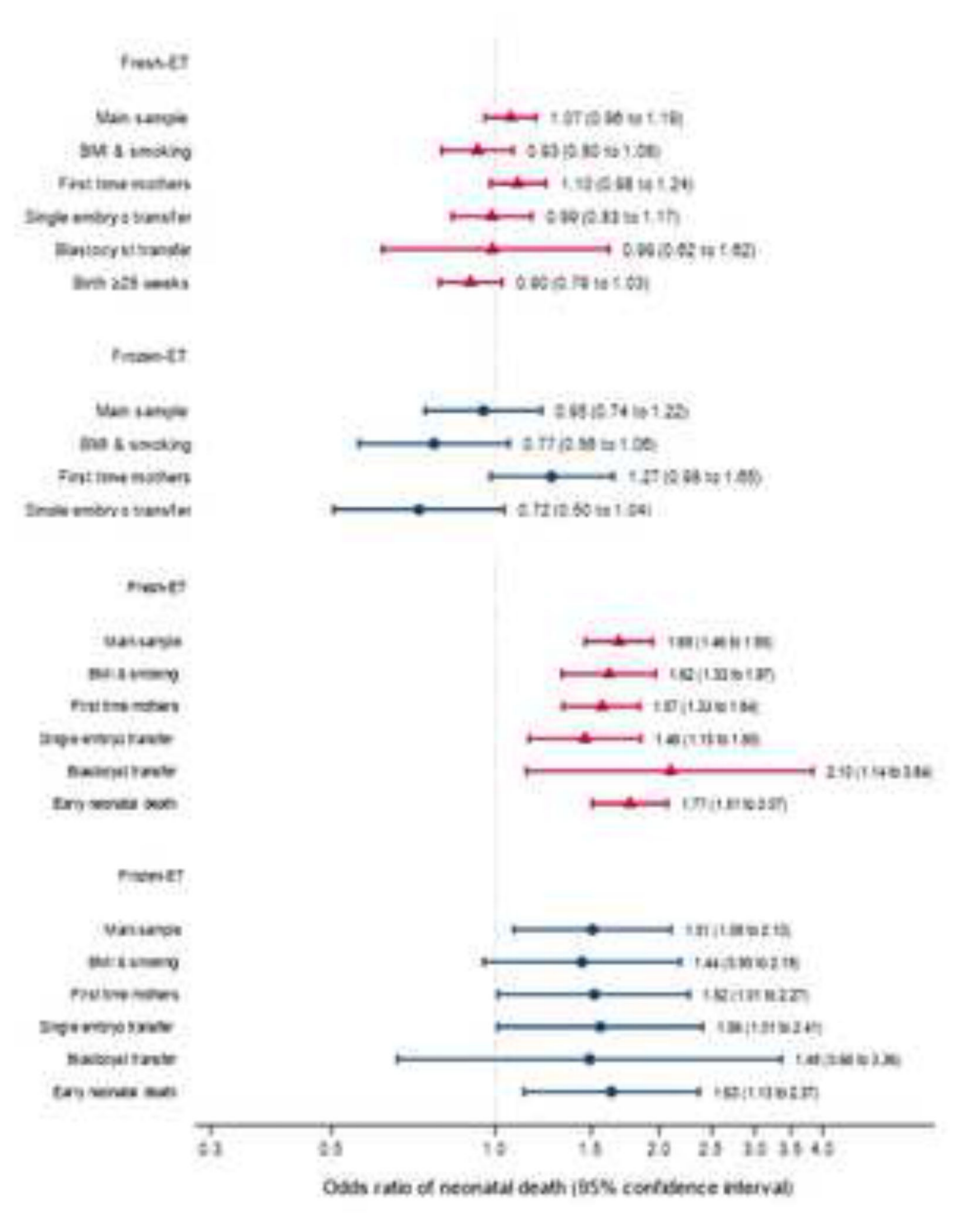
Odds ratios with 95% confidence intervals for stillbirth (upper panel) and neonatal death (lower panel) according to conception method and analysis sample. Reference is conception without medical assistance. Adjusted for maternal age, parity (if applicable), country, year of birth. Analyses in the sample titled “BMI & smoking” are additionally adjusted for maternal body mass index, height and smoking during pregnancy.

We found no clear association between conception method and risk of stillbirth, while neonatal mortality was higher after both fresh and frozen-ET compared to singletons born without medical assistance (Table 2). Adjustment for available confounders had little impact on the associations. Results from sensitivity analyses supported those from the main analyses (Figure 2, Supplemental Tables 2-3). There was some evidence of heterogeneity in the point estimates for stillbirth, where both fresh and frozen transfers in Sweden had lower risk than pregnancies conceived without medical assistance, but with no conclusive evidence of higher risk in Denmark and Norway (Supplemental Table 4). For neonatal mortality, associations were similar across all countries.

In analyses according to gestational age, the absolute risk of stillbirth was highest in term (37-41 weeks) gestations, when most deliveries occurred (Table 3 and Supplemental Figure 1). For singletons born after fresh-ET, risk of stillbirth was higher than for singletons born without medical assistance in 22-27 weeks gestation, and thereafter similar, although with wide confidence intervals. Risk in pregnancies after frozen-ET did not clearly differ from that in pregnancies conceived without medical assistance at any gestational ages, but precision was low. Neonatal mortality (Table 3, Supplemental Figure 2) was highest for live births at 22-27 weeks (fresh-ET 24.8%, frozen-ET 21.0%, and without medical assistance 25.3%) and declined steeply with increasing gestational age to the lowest observed risk for term live births (37-41 weeks, fresh-ET and frozen-ET 0.06% and singletons conceived without medical assistance 0.07%). However, for each gestational period the risk of neonatal death was similar for fresh and frozen-ET compared to singletons conceived without medical assistance, apart from a higher risk post term for fresh-ET, although with wide confidence intervals.

## Discussion

### Principal findings

Compared to singletons born without medical assistance, singletons conceived after fresh and frozen-ET had an overall similar risk of stillbirth, but a higher risk of neonatal death. Apart from a higher risk of stillbirth in pregnancies after fresh-ET in weeks 22-27, we found no clear differences in associations for fresh and frozen-ET. The higher risk of neonatal death after both fresh and frozen-ET might be attributed to a higher risk of preterm birth in ART pregnancies.

### Comparisons to other studies

The lack of association between conception method and risk of stillbirth in our study is in contrast to results from a population-based study from the Netherlands, where a nearly doubled risk of stillbirth was found for any ART (n=19,896) compared to pregnancies conceived without medical assistance(n=999,050, OR 1.94, 95% CI 1.54-2.44).(15) A meta-analysis comparing 68,274 ART-conceived and 3,570,990 conceived without medical assistance, mainly from cohort studies, also found higher odds of stillbirth after ART (OR 1.41, 95%CI 1.20-1.65).(14) Previous observations from the Nordic countries, overlapping our study population with births from 1988 to 2007, indicated similar risk of stillbirth for both frozen-ET (n=6,647) and fresh-ET (n= 42,242) compared to conceptions without medical assistance (n=288,542).(23)

Our results are consistent with the limited number of previous studies on conception method and neonatal mortality, showing higher neonatal mortality after any ART,(15, 19, 23) but do not support previous observations of higher neonatal mortality after frozen-ET compared to fresh-ET.(21–23)

We are not aware of previous studies assessing whether pregnancies after fresh and frozen-ET are more vulnerable to stillbirth or neonatal death at specific gestational ages. An Australian study compared pregnancies after any ART (n=15,416) to pregnancies conceived without medical assistance(n=391,952) and found lower perinatal mortality <32 weeks.(20) However, they did not use a ‘fetuses at risk’ approach and further differed from our study by including births and terminations ≥20 weeks gestation. A Danish study showed higher risk of stillbirth at term after ART compared to pregnancies conceived without medical assistance, but included only uncomplicated pregnancies, thereby increasing the risk of selection bias.(35) Rather our findings support a previous Nordic study, where the study population overlaps ours until 2007,(19) and a higher risk of stillbirth during week 22-27 was found for any ART (n=62,485) compared to pregnancies conceived without medical assistance(n=362,798). Our study adds to this observation by indicating that the higher risk may only apply to fresh-ET. This finding is also consistent with results from two randomized trials comparing fresh- and frozen-ET, where a lower risk of second trimester stillbirth was found in pregnancies after frozen-ET.(36, 37)

In the early years of our study period, slow freeze was the standard method of cryopreservation but was gradually replaced by vitrification from around 2008. In parallel culture duration shifted from cleavage stage to blastocyst before embryo freezing. A comparison of singletons from slow-frozen cleavage stage embryos vs vitrified blastocysts in Denmark and Sweden showed no difference in perinatal or neonatal death, but with very limited precision(38). Our sensitivity analyses restricted to blastocyst transfers are in line with those observations.

We had no data on endometrial preparation in frozen-ET. Previous studies indicate lower risk of hypertensive disorders in pregnancies with no endometrial preparation (natural cycles),(39) but similar risks of both stillbirth and neonatal death in pregnancies conceived in natural, programmed and stimulated frozen-ET cycles.(40) (39)

### Implications

Despite the higher risk of adverse perinatal outcomes in ART pregnancies, including preterm birth, low birthweight, and placental complications as shown previously in this study population,(18, 29, 41) we found no higher risk of stillbirth in ART-conceived pregnancies. In contrast, neonatal mortality was higher after both fresh and frozen-ET compared with singletons conceived without medical assistance, but the higher risk attenuated in analyses according to gestational age. Preterm birth is a strong risk factor for neonatal death,(42) and our results suggest that the higher risk of preterm birth after ART,(18) may strongly contribute to their higher risk of neonatal death. Identifying women at risk of preterm birth may therefore provide an important means of reducing neonatal mortality associated with ART, as well as neonatal morbidity and other long term, adverse health consequences associated with preterm birth.(16, 42)

In the country specific estimations (Supplemental table 6) we found that Sweden showed an overall lower prevalence and risk of stillbirth among fresh and frozen-ET pregnancies compared to pregnancies conceived without medical assistance. The underlying mechanisms for this may be many, but in 2003 Sweden introduced a policy of single embryo transfer, and it would be interesting to know if such a policy has the potential to improve the overall perinatal outcomes as well as preventing cases of stillbirth.

Early in our study period, women with ART conceptions had more antenatal visits than women conceiving without medical assistance.(43, 44) However, the existing antenatal care programs in the Nordic countries do not target ART conceptions or subfertility directly, including screening for placental complications.

Future studies should investigate whether indicators of infertility may improve risk stratification of pregnancies beyond current guidelines.

For infertile couples and clinicians, the similar associations for fresh-ET and frozen-ET do not give reason to prefer one method over the other to prevent stillbirth or neonatal death.

### Strengths and limitations

To our knowledge this is the largest study so far of stillbirth and neonatal mortality after fresh and frozen-ET compared to singletons conceived without medical assistance, including an additional 51,000 ART singletons born 2008-2014/2015 compared to previous Nordic registry studies.(19, 23) The sensitivity analyses collectively strengthen validity and do not support that the results could be attributed to other treatment characteristics, changes in registration practice, or to maternal factors such as BMI and smoking. However, residual confounding from socioeconomic status or causes of infertility cannot be excluded. Several causes of female infertility, such as endometriosis and polycystic ovarian syndrome, are associated with adverse perinatal outcomes and may contribute to perinatal loss, directly or through higher risk of preterm birth (45–47). Data on causes of infertility were too limited to allow further analyses. In Nordic clinics, male factor has been the main indication for ICSI for most of the study period(4), and previous Nordic studies have used ICSI fertilization as a proxy for male factor infertility(41, 48). However, the low number of events did not permit further stratification on fertilization method. Several studies show that perinatal outcomes are affected by the composition of culture media(49, 50). Unfortunately, we had no data on which culture media were used, but these have varied over time and between clinics. Hence, differences in exposure to culture media between fresh and frozen embryos cannot be excluded.

The national identity number ensured reliable data linkage and reporting to the registries is mandatory in all participating countries(51). Data on conception method and pregnancy outcome are collected independently, from ART clinics and delivery institutions, respectively, thereby minimizing differential misclassification.

Because gestational age is an intermediate factor between ART-conception and neonatal death, interpretation of our analyses of neonatal death according to gestational age requires careful consideration(52). In terms of prediction(16), our results suggest that infants conceived after fresh- or frozen-ET are equally vulnerable to the impact of preterm birth as pregnancies conceived without medical assistance. In terms of mechanisms, they suggest that the higher neonatal mortality might be attributed to the higher risk of preterm birth in ART-conceived pregnancies. Importantly, this interpretation depends on no unmeasured confounding between gestational age and neonatal death,(53) and no differential selection forces of ART on fetal survival(34). These assumptions are likely not met as for example placental complications could be common causes of preterm birth and neonatal death, but also be affected by ART-conception(29, 54). Although we could not determine the causes and course of delivery, higher proportions of caesarean section for ART compared to natural conception in cases of neonatal death, suggest that ART-conceived pregnancies were more often delivered on medical indication.

Despite including data from three countries, statistical power was limited. Estimates according to gestational age had low precision due to the stratification itself, but also because stillbirths often had missing data on other factors, including gestational age.

It should be noted that neonatal death and stillbirth rates are low in all Nordic countries, even when compared to other high-income countries(27, 55, 56). Further, due to public funding of ART treatment combined with widespread availability in all Nordic countries, the couples’ socioeconomic background may influence access to ART less than in other settings. Currently, births after ART-conception comprise around 5% of birth cohorts in the Nordic countries,(57, 58) but despite this high availability of treatment, long duration of infertility among treated couples suggests that treatment indications are not unusually liberal.(59) Lastly, perinatal care is standardized and free of charge, with high adherence among pregnant women. These similarities between the Nordic countries strengthen the arguments to pool data across countries (51), but may limit generalizability to other societies.

### Conclusion

Our results suggest that singletons conceived by fresh and frozen embryo transfer are not at higher overall risk of stillbirth compared with pregnancies conceived without medical conceptions, though singletons after fresh-ET may be at higher risk in gestational week 22-27. Both types of ART-conception carry a higher risk of neonatal death, possibly mediated by preterm birth. The similarity of results between fresh and frozen-ET treatment, indicate that for couples in need of ART, reassurance can be provided that a frozen embryo is unlikely to increase risk of stillbirth and neonatal mortality compared with fresh transfer (and vice versa).

## Supplementary Material

Supplementary material

Supplementary Figures

## Figures and Tables

**Table 1:** Characteristics of the total study population, and for stillbirths and neonatal deaths (0-27 days) separately

Characteristic ^1^	Total population			Stillbirths			Neonatal Deaths		
	Natural conception	Fresh-ET	Frozen-ET	Natural conception	Fresh-ET	Frozen-ET	Natural conception	Fresh-ET	Frozen-ET
Participants ^2^	4,494,117 (97.9)	78,642 (1.7)	18,094 (0.4)	16,701 (0.37)	357 (0.45)	65 (0.36)	7,439 (0.17)	210 (0.27)	36 (0.20)
Mean maternal age in years (SD)	29.6 (4.8)	33.8 (4.2)	34.3 (4.1)	29.9 (5.2)	33.5 (4.3)	34.2 (3.8)	29.3 (5.0)	33.9 (4.1)	34.9 (4.7)
Primiparity	1,303,268 (51.3)	59,188 (75.3)	10,493 (58.0)	9,615 (57.6)	302 (84.6)	57 (87.7)	4,284 (57.6)	163 (77.6)	24 (66.7)
Mean maternal BMI in kg/m^2^ (SD)	24.2 (4.5)	24.2 (4.1)	24.2 (4.0)	25.6 (5.3)	25.5 (4.6)	25.3 (3.7)	25.1 (5.2)	25.2 (4.4)	25.7 (4.2)
Maternal smoking ^3^	451,931 (12.0)	4,070 (5.7)	542 (3.2)	2,170 (17.9)	32 (10.7)	0	948 (17.0)	13 (7.8)	-- ^4^
Small for gestational age ^3, 5^	165,729 (3.7)	4,224 (5.4)	532 (3.0)	4,336 (26.0)	87 (24.4)	12 (18.5)	1,889 (25.4)	49 (23.3)	9 (25.0)
Large for gestational age ^3, 6^	200,256 (4.5)	2,841 (3.6)	1,172 (6.5)	542 (3.3)	6 (1.7)	5 (7.7)	405 (5.4)	12 (5.7)	-- ^4^
Preterm birth <37 weeks ^3^	221,006 (5.0)	6,335 (8.1)	1,198 (6.6)	8,522 (55.0)	217 (62.4)	32 (49.2)	4,474 (60.1)	158 (75.2)	26 (72.2)
Very preterm birth <32 weeks ^3^	30,899 (0.70)	1,179 (1.50)	216 (1.2)	4,924 (31.8)	143 (41.1)	22 (33.9)	3,086 (41.5)	128 (61.0)	21 (58.3)
Extremely preterm birth <28 weeks ^3^	8,123 (0.18)	355 (0.45)	81 (0.45)	2,664 (17.2)	110 (31.6)	16 (24.6)	2,196 (29.5)	99 (47.1)	17 (47.2)
Malformations, any	188,030 (4.2)	4,541 (5.7)	862 (4.8)	756 (4.5)	11 (3.1)	3 (4.6)	2,531 (34.0)	57 (27.1)	7 (19.4)
Sex, girls	2,183,621 (48.6)	38,404 (48.8)	8,853 (48.9)	7,487 (44.8)	145 (40.6)	42 (64.6)	3,165 (42.6)	98 (46.7)	21 (58.3)
Induction of delivery	577,707 (12.9)	14,841 (18.9)	4,524 (25.0)	9,580 (57.4)	200 (56.0)	32 (49.2)	960 (12.9)	28 (13.3)	4 (11.1)
Caesarean section	679,474 (15.1)	19,943 (25.4)	5,143 (28.4)	1,595 (9.6)	34 (9.5)	5 (7.7)	3,288 (44.2)	115 (54.8)	18 (50.0)
Placental disorders ^7^	222,752 (5.0)	6,543 (8.3)	1,601 (8.9)	2,110 (12.6)	48 (13.5)	5 (7.7)	1,222 (16.4)	48 (22.9)	-- ^4^
Fertilization by ICSI ^3^	-	32,377 (42.0)	6,631 (40.2)	-	132 (37.7)	24 (44.4)	-	72 (34.8)	5 (16.1)
Blastocyst culture ^3^	-	4,462 (5.7)	3,762 (20.8)	-	18 (5.0)	9 (13.9)	-	11 (6.3)	6 (19.4)
Single embryo transfer ^3^	-	37,192 (53.7)	11,620 (72.2)	-	143 (44.4)	30 (57.7)	-	72 (39.3)	21 (72.4)

1 Number of observations (%), with percentages calculated from the total number of participants in each column, unless otherwise specified.

2 Percentages sum up to 100 across conception method in Total population, but are occurrences of stillbirth and neonatal death within conception method (% of Total population).

3 Percentages were calculated after excluding observations with missing data.

4 Number of observations could not be presented due to data privacy.

5 Birthweight <-22% and

6 birthweight >+22% of expected mean according to sex and gestational age, as defined in Marsal K, et al. Acta Paediatr 1996;85:843-8.

7 Placental abruption, placenta previa and hypertensive disorders in pregnancy, as defined in Petersen et al, Am J Obstet Gynecol 2020; 223(2): 226.e19.

Abbreviations: BMI – body mass index, ET – embryo transfer, SD – standard deviation

**Table 2:** Risk of stillbirth and neonatal death by conception method in main analyses samples

	Numbers	Risk ^1^, %	RD (95%CI) ^1^, *pp*	RD (95% CI) ^2^, *pp*	OR (95% CI) ^1^	OR (95% CI) ^2^
**Stillbirth**						
Pregnancies without medical assistance	4,494,117	0.37	0	0	1	1
Fresh-ET	78,642	0.45	0.08 (0.03 to 0.13)	0.02 (-0.02 to 0.06)	1.22 (1.10 to 1.36)	1.05 (0.94 to 1.17)
Frozen-ET	18,094	0.36	-0.01 (-0.10 to 0.07)	-0.03 (-0.11 to 0.06)	0.96 (0.75 to 1.23)	0.92 (0.72 to 1.18)
**Neonatal death (0-27 days)**						
Pregnancies without medical assistance	4,414,705	0.17	0	0	1	1
Fresh-ET	78,095	0.27	0.10 (0.06 to 0.14)	0.11 (0.07 to 0.15)	1.60 (1.39 to 1.84)	1.69 (1.46 to 1.95)
Frozen-ET	17,990	0.20	0.03 (-0.03 to 0.10)	0.08 (0.00 to 0.16)	1.18 (0.85 to 1.65)	1.51 (1.08 to 2.10)

Abbreviations: RD – risk difference, *pp* – percentage points, Adj. – adjusted, CI – confidence interval, OR – odds ratio.

1 Unadjusted.

2 Adjusted for maternal age, parity, country, offspring year of birth

**Table 3:** Risk of stillbirth and neonatal death according to conception method and gestational age at birth.

	Stillbirth					Neonatal deaths				
	Deaths, n	Pregnancies at risk, n	Risk ^1^, %	OR (95% CI) ^1^	OR (95% CI) ^2^	Deaths, n	Live births, n	Risk ^1^, %	OR (95% CI) ^1^	OR (95% CI) ^2^
**Gestational age 22-27 weeks**										
Pregnancies without medical assistance	2,664	2,454,283	0.11	1	1	2,196	8,692	25.3	1	1
Fresh-ET	110	51,333	0.21	2.00 (1.64 to 2.44)	1.85 (1.51 to 2.27)	99	400	24.8	0.97 (0.77 to 1.23)	1.04 (0.82 to 1.33)
Frozen-ET	16	13,991	0.11	1.05 (0.64 to 1.73)	1.12 (0.68 to 1.86)	17	81	21.0	0.78 (0.45 to 1.35)	1.04 (0.60 to 1.80)
**Gestational age 28-31 weeks**										
Pregnancies without medical assistance	2,260	4,430,654	0.05	1	1	890	19,599	4.6	1	1
Fresh-ET	33	78,158	0.04	0.83 (0.59 to 1.17)	0.77 (0.54 to 1.09)	29	774	3.8	0.75 (0.45 to 1.23)	1.06 (0.66 to 1.72)
Frozen-ET	6	17,999	0.03	0.65 (0.29 to 1.46)	0.71 (0.32 to 1.60)	4	127	3.2	0.56 (0.15 to 2.06)	1.00 (0.29 to 3.46)
**Gestational age 32-36 weeks**										
Pregnancies without medical assistance	3,598	4,407,878	0.08	1	1	1,388	184,911	0.8	1	1
Fresh-ET	74	77,334	0.10	1.17 (0.93 to 1.48)	1.16 (0.92 to 1.47)	30	5,051	0.6	0.77 (0.52 to 1.15)	1.09 (0.73 to 1.63)
Frozen-ET	10	17,864	0.06	0.69 (0.37 to 1.28)	0.74 (0.40 to 1.38)	5	967	0.5	0.67 (0.26 to 1.73)	0.99 (0.38 to 2.58)
**Gestational age 37-41 weeks**										
Pregnancies without medical assistance	6,416	4,217,771	0.15	1	1	2,142	3,012,703	0.07	1	1
Fresh-ET	121	72,178	0.17	1.10 (0.91 to 1.32)	0.91 (0.76 to 1.09)	32	53,201	0.06	0.85 (0.60 to 1.20)	0.95 (0.66 to 1.35)
Frozen-ET	30	16,882	0.18	1.17 (0.81 to 1.67)	1.05 (0.73 to 1.51)	7	11,660	0.06	0.85 (0.40 to 1.78)	1.14 (0.53 to 2.40)
**Gestational age 42-44 weeks**										
Pregnancies without medical assistance	560	334,559	0.17	1	1	311	332,933	0.1	1	1
Fresh-ET	8	4,413	0.18	1.09 (0.49 to 2.40)	0.85 (0.39 to 1.87)	8	4,396	0.2	1.95 (0.97 to 3.94)	2.08 (1.01 to 4.30)
Frozen-ET	3	1,332	0.23	1.40 (0.38 to 5.13)	1.35 (0.37 to 4.89)	0	1,324	0	-	-

Abbreviations: n - numbers, CI – confidence interval, OR – odds ratio.

1 Unadjusted.

2 Adjusted for maternal age, parity, country, offspring year of birth

## Data Availability

The data underlying this article cannot be shared publicly due to the data protection and privacy of individuals that participated in the study. The procedures for data access are described Opdahl S, et al. Data resource profile: the Committee of Nordic Assisted Reproductive Technology and Safety (CoNARTaS) cohort. Int J Epidemiol. 2020;49(2):365-366f.

## Capsule

Overall, risk of stillbirth was similar after fresh and frozen embryo transfer compared to natural conception, whereas neonatal mortality was higher, possibly due to preterm birth.
