# Supplementary material for: Risk of stillbirth and neonatal death in singletons born after fresh and frozen embryo transfer. Cohort study from the Committee of Nordic Assisted Reproduction Technology and Safety
Source: Fertil Steril. Author manuscript; Available in PMC 2023 Nov 20. (PMC7615319; doi:10.1016/j.fertnstert.2022.10.020)
Supplement: Supplementary material [file EMS189986-supplement-Supplementary_material.docx]

**Supplemental Materials**

Supplemental Methods. Fetuses at risk as the denominator for neonatal mortality.

Supplemental Table 1. Causes of neonatal death (0-27 days) according to conception method. Categorized according to the World Health Organization application of ICD-10 to deaths during the perinatal period (ICD-PM)

Supplemental Table 2. Risk of stillbirth by conception method: sensitivity analyses in subsamples accounting for maternal and treatment characteristics, as well as outcome definitions

Supplemental Table 3. Risk of neonatal death (0-27 days) by conception method: sensitivity analyses in subsamples accounting for maternal and treatment characteristics, as well as outcome definitions

Supplemental Table 4. Risk of stillbirth and neonatal death (0-27 days) according to conception method. Country-specific analyses of pregnancies and births with gestational age 28-44 weeks.

Supplemental Table 5. Risk of death according to conception method and gestational age at birth, separate analyses for periods 1988-2007 and 2008-2014/15

Supplemental Table 6. Risk of neonatal death (0-27 days) according to conception method. Comparison of associations using ongoing pregnancies vs live births as the denominator

Supplemental Figure 1. Risk of stillbirth according to conception method and gestational age at birth

Supplemental Figure 2. Risk of neonatal death according to conception method and gestational age at birth

| **Supplemental Table 1. Causes of neonatal death 0-27 days according conception method. Categorized according to the World Health Organization application of ICD-10 to deaths during the perinatal period (ICD-PM) ^1^** | | | | |
| --- | --- | --- | --- | --- |
| **Cause of death** | **ICD-10 codes** | **ICD-9 codes ^2^** | **Natural conception** | **ART conception** |
| N1 Congenital malformations, deformations and chromosomal abnormalities | Q00-Q99 | 740-759 | 2,602 (35.0) | 48 (19.5) |
| N3 Birth trauma ^3^  N4 Complications of intrapartum events ^3^ | P10-P15, P20-P21 | 767, 768 | 661 (8.9) | 21 (8.5) |
| N6 Infection | A33, A50, G00-G09, P23,  P35-39 | 090, 320-326, 770.0, 771 | 378 (5.1) | 9 (3.7) |
| N7 Respiratory and cardiovascular disorders | P22, P24-P29 | 769, 770 | 649 (8.7) | 17 (6.9) |
| N5 Convulsions and disorders of cerebral status ^3^ N8 Other neonatal conditions ^3^ | P50-P61, P70-P78, P80-P83, P90-94 | 772-778  779.0-779.4 | 405 (5.4) | 23 (9.3) |
| N2 Disorders related to fetal growth ^3^  N9 Low birth weight and prematurity ^3^ | P05, P07, P08 | 764-766 | 703 (9.5) | 48 (19.5) |
| N11 Neonatal death of unspecified cause | P96 | 779.5-779.9 | 42 (0.6) | 0 (0) |
| Maternal conditions | P00-P04 | 760-763 | 1,208 (16.2) | 60 (24.4) |
| Causes not included in ICD-PM (i.e., not specific to the perinatal period) | All other specified codes | All other specified codes | 791 (10.6) | 20 (8.1) |
| All neonatal deaths |  |  | 7,439 (100) | 246 (100) |
| ^1^ Flenady et al. Semin Fetal Neonatal Med 2017; 22(3):176-185. ^2^ Adapted by the authors. ICD-9 was used until 1995 in Norway and 1996 in Sweden. ^3^ ICD-PM categories are combined due to data privacy rules | | | | |

| **Supplemental Table 2. Risk of stillbirth by conception method: sensitivity analyses in subsamples accounting for maternal and treatment characteristics, as well as outcome definitions** | | | | | | |
| --- | --- | --- | --- | --- | --- | --- |
|  | **Numbers** | **Risk ^1^, %** | **RD (95% CI) ^1^, *pp*** | **RD (95% CI) ^2^, *pp*** | **OR (95% CI) ^1^** | **OR (95% CI) ^2^** |
| **Sensitivity analysis 1: Available data on maternal BMI and smoking ^3^** | | | | | | |
| Pregnancies without medical assistance | 2,561,775 | 0.30 | 0 | 0 | 1 | 1 |
| Fresh-ET | 53,331 | 0.34 | 0.03 (-0.02 to 0.08) | -0.02 (0.06-0.02) | 1.11 (0.96 to 1.29) | 0.93 (0.80 to 1.08) |
| Frozen-ET | 14,389 | 0.27 | -0.03 (-0.12 to 0.05) | -0.07 (-0.14 to 0.01) | 0.89 (0.65 to 1.22) | 0.77 (0.56 to 1.06) |
| **Sensitivity analysis 2: First time mothers ^4^** | | | | | | |
| Pregnancies without medical assistance | 2,303,268 | 0.42 | 0 | 0 | 1 | 1 |
| Fresh-ET | 59,188 | 0.51 | 0.09 (0.03 to 0.15) | 0.04 ^4^ (-0.01 to 0.09) | 1.22 (1.09 to 1.37) | 1.10 ^4^ (0.98 to 1.24) |
| Frozen-ET | 10,493 | 0.54 | 0.13 (-0.02 to 0.27 | 0.11 ^4^ (-0.03 to 0.25) | 1.30 (1.00 to 1.70) | 1.27 ^4^ (0.98 to 1.65) |
| **Sensitivity analysis 3: Single embryo transfer** | | | | | | |
| Pregnancies without medical assistance | 4,494,117 | 0.37 | 0 | 0 | 1 | 1 |
| Fresh-ET | 37,192 | 0.38 | 0.01 (-0.05 to 0.08) | -0.01 (-0.07 to 0.01) | 1.03 (0.88 to 1.22) | 0.99 (0.83 to 1.17) |
| Frozen-ET | 11,620 | 0.26 | -0.11 (-0.21 to -0.02) | -0.1 (-0.20 to -0.00) | 0.69 (0.48 to 0.99) | 0.72 (0.50 to 1.04) |
| **Sensitivity analysis 4: Blastocysts transfer** | | | | | | |
| Pregnancies without medical assistance | 2,455,640 | 0.35 | 0 | 0 | 1 | 1 |
| Fresh-ET | 4,458 | 0.38 | 0.03 (-0.15 to 0.21) | -0.008 (-0.17 to 0.16) | 1.09 (0.67to 1.78) | 1.00 (0.62 to 1.62) |
| Frozen-ET | 3,762 | 0.24 | -0.11 (-0.27 to 0.05) | -0.12 (-0.3 to 0.03) | 0.68 (0.35 to 1.32) | 0.65 (0.34 to 1.26) |
| **Sensitivity analysis 5: Births ≥28 weeks gestation** | | | | | | |
| Pregnancies without medical assistance | 4,485,994 | 0.35 | 0 | 0 | 1 | 1 |
| Fresh-ET | 78,287 | 0.45 | 0.00 (-0.04 to 0.04) | -0.03 (-0.07 to 0.01) | 1.01 (0.89 to 1.14) | 0.90 (0.79 to 1.03) |
| Frozen-ET | 18,013 | 0.35 | -0.04 (-0.12 to 0.03) | -0.03 (-0.11 to 0.04) | 0.87 (0.65 to 1.15) | 0.89 (0.67 to 1.18) |
| Abbreviations: RD – risk difference, pp – percentage points, Adj. – adjusted, CI – confidence interval, OR – odds ratio.  ^1^ Unadjusted. ^2^ Adjusted for maternal age, parity, country, year of birth ^3^ Additionally adjusted for maternal BMI and smoking status. ^4^ Not adjusted for parity | | | | | | |

| **Supplemental Table 3. Risk of neonatal death (0-27 days) by conception method: sensitivity analyses in subsamples accounting for maternal and treatment characteristics, as well as outcome definitions** | | | | | | |
| --- | --- | --- | --- | --- | --- | --- |
|  | **Numbers** | **Risk ^1^, %** | **RD (95% CI) ^1^, *pp*** | **RD (95% CI) ^2^, *pp*** | **OR (95% CI) ^1^** | **OR (95% CI) ^2^** |
| **Sensitivity analysis 1: Available data on maternal BMI and smoking ^3^** | | | | | | |
| Pregnancies without medical assistance | 2,548,239 | 0.13 | 0 | 0 | 1 | 1 |
| Fresh-ET | 53,059 | 0.20 | 0.08 (0.04 to 0.11) | 0.08 (0.04 to 0.12) | 1.60 (1.32 to 1.94) | 1.62 (1.33 to 1.97) |
| Frozen-ET | 14,326 | 0.16 | 0.03 (-0.03 to 0.10) | 0.06 (-0.02 to 0.13) | 1.26 (0.83 to 1.90) | 1.44 (0.95 to 2.19) |
| **Sensitivity analysis 2: First time mothers ^4^** | | | | | | |
| Pregnancies without medical assistance | 2,258,213 | 0.19 | 0 | 0 | 1 | 1 |
| Fresh-ET | 58,739 | 0.28 | 0.09 (0.04 to 0.13) | 0.11 (0.06 to 0.15) | 1.46 (1.25 to 1.71) | 1.57 (1.33 to 1.84) |
| Frozen-ET | 10,413 | 0.23 | 0.04 (-0.05 to 0.13) | 0.10 (-0.02 to 0.21) | 1.22 (0.81 to 1.82) | 1.52 (1.01 to 2.27) |
| **Sensitivity analysis 3: Single embryo transfer** | | | | | | |
| Pregnancies without medical assistance | 4,414,705 | 0.17 | 0 | 0 | 1 | 1 |
| Fresh-ET | 36,992 | 0.19 | 0.03 (-0.02 to 0.07) | 0.08 (0.02 to 0.13) | 1.15 (0.91 to 1.46) | 1.46 (1.15 to 1.85) |
| Frozen-ET | 11,577 | 0.18 | 0.01 (-0.06 to 0.09) | 0.09 (-0.02 to 0.20) | 1.08 (0.70 to 1.66) | 1.56 (1.09 to 2.41) |
| **Sensitivity analysis 4: Blastocysts transfer** | | | | | | |
| Pregnancies without medical assistance | 2,430,870 | 0.14 | 0 | 0 | 1 | 1 |
| Fresh-ET | 4,423 | 0.25 | 0.11 (-0.04 to 0.25) | 0.15 (-0.02 to 0.32) | 1.76 (0.97 to 3.22) | 2.10 (1.14 to 3.84) |
| Frozen-ET | 3,742 | 0.16 | 0.02 (-0.11 to 0.14) | 0.07 (-0.1 to 0.23) | 1.13 (0.50 to 2.55) | 1.49 (0.66 to 3.36) |
| **Sensitivity analysis 5: Early neonatal death (0-6 days after birth)** | | | | | | |
| Pregnancies without medical assistance | 4,414,705 | 0.13 | 0 | 0 | 1 | 1 |
| Fresh-ET | 78,095 | 0.22 | 0.09 (0.05 to 0.11) | 0.10 (0.06 to 0.13) | 1.70 (1.45 to 1.98) | 1.77 (1.51 to 2.07) |
| Frozen-ET | 17,990 | 0.16 | 0.03 (-0.02 to 0.09) | 0.08 (0.00 to 0.15) | 1.27 (0.88 to 1.83) | 1.63 (1.13 to 2.37) |
| Abbreviations: RD – risk difference, pp – percentage points, Adj. – adjusted, CI – confidence interval, OR – odds ratio, Ref. – reference.  ^1^ Unadjusted. ^2^ Adjusted for maternal age, parity, country, year of birth ^3^ Additionally adjusted for maternal BMI and smoking status. ^4^ Not adjusted for parity | | | | | | |

| **Supplemental Table 4. Risk of stillbirth and neonatal death (0-27 days) according to conception method. Country-specific analyses of pregnancies and births with gestational age 28-44 weeks.** | | | | | | | | | | |  |
| --- | --- | --- | --- | --- | --- | --- | --- | --- | --- | --- | --- |
|  | **Stillbirth** | | | | | **Neonatal death** | | | | |  |
|  | **Deaths, n** | **Pregnancies at risk, n** | **Risk ^1^, %** | **OR (95% CI) ^1^** | **OR (95% CI) ^2^** | **Deaths, n** | **Live births, n** | **Risk ^1^, %** | **OR (95% CI) ^1^** | **OR (95% CI) ^2^** |  |
| **Denmark** | | | |  |  |  |  |  |  |  |  |
| Pregnancies without medical assistance | 2,827 | 983,625 | 0.29 | 1 | 1 | 1,806 | 977,754 | 0.18 | 1 | 1 |  |
| Fresh-ET | 93 | 25,106 | 0.37 | 1.29 (1.05 to 1.59) | 1.16 (0.94 to 1.46) | 82 | 25,041 | 0.33 | 1.78 (1.42 to 2.23) | 1.92 (1.52 to 2.42) |  |
| Frozen-ET | 13 | 3,356 | 0.39 | 1.35 (0.78 to 2.34) | 1.46 (0.84 to 2.53) | 8 | 3,347 | 0.24 | 1.29 (0.64 to 2.60) | 1.62 (0.80 to 3.28) |  |
| **Norway** | | | |  |  |  |  |  |  |  |  |
| Pregnancies without medical assistance | 3,403 | 1,196,200 | 0.28 | 1 | 1 | 1,996 | 1,193,617 | 0.17 | 1 | 1 |  |
| Fresh-ET | 53 | 16,537 | 0.32 | 1.13 (0.86 to 1.48) | 1.05 (0.80 to 1.39) | 54 | 16,551 | 0.33 | 1.97 (1.49 to 2.60) | 2.13 (1.61 to 2.83) |  |
| Frozen-ET | 10 | 3,282 | 0.30 | 1.07 (0.57 to 2.00) | 1.15 (0.62 to 2.16) | 7 | 3,283 | 0.21 | 1.27 (0.60 to 2.71) | 1.60 (0.75 to 3.41) |  |
| **Sweden** | | | |  |  |  |  |  |  |  |  |
| Pregnancies without medical assistance | 6,604 | 2,250,829 | 0.29 | 1 | 1 | 3,637 | 2,243,334 | 0.16 | 1 | 1 |  |
| Fresh-ET | 90 | 36,515 | 0.25 | 0.84 (0.68 to 1.03) | 0.72 (0.58 to 0.89) | 74 | 36,503 | 0.20 | 1.25 (0.99 to 1.58) | 1.34 (1.06 to 1.70) |  |
| Frozen-ET | 26 | 11,361 | 0.23 | 0.78 (0.53 to 1.14) | 0.72 (0.49 to 1.06) | 21 | 11,360 | 0.18 | 1.14 (0.74 to 1.76) | 1.43 (0.93 to 2.22) |  |
| Abbreviations: n - numbers, CI – confidence interval, OR – odds ratio.  ^1^ Unadjusted. ^2^ Adjusted for maternal age, parity, country, offspring year of birth. Likelihood ratio tests of interaction in adjusted models: p (stillbirth) = 0.03, p (neonatal death) = 0.16 | | | | | | | | | | |  |

| **Supplemental Table 5. Risk of death according to conception method and gestational age at birth, separate analyses for periods 1988-2007 and 2008-2014/15** | | | | | | | | | | | | | |
| --- | --- | --- | --- | --- | --- | --- | --- | --- | --- | --- | --- | --- | --- |
|  |  | **Stillbirths 1988-2007** | | | | | | **Stillbirths 2008-2014/15** | | | | | |
| **Gestational age** | **Conception method** | **Deaths, n** | **Pregnancies at risk, n** | **Risk ^1^, %** | **OR ^1^** | **OR ^2^** | **95% CI ^2^** | **Deaths, n** | **Pregnancies**  **at risk, n** | **Risk ^1^, %** | **OR ^1^** | **OR ^2^** | **95% CI ^2^** |
| **22-27 weeks ^3^** | Pregnancies without medical assistance | 1,260 | 1,005,099 | 0.13 | 1 | 1 | Ref. | 1,404 | 1,449,184 | 0.10 | 1 | 1 | Ref. |
|  | ART | 38 | 14,247 | 0.27 | 2.15 | 2.19 | 1.56 to 3.09 | 88 | 51,077 | 0.17 | 1.80 | 1.53 | 1.22 to 1.92 |
|  | *Fresh-ET* | *35* | *12,912* | *0.27* | *2.19* | *2.21* | *1.55 to 3.16* | *75* | *38,421* | *0.20* | *2.05* | *1.69* | *1.32 to 2.16* |
|  | *Frozen-ET* | *3* | *1,335* | *0.22* | *1.80* | *1.99* | *0.62 to 6.40* | *13* | *12,656* | *0.10* | *1.06* | *0.98* | *0.56 to 1.72* |
| **28-31 weeks** | Pregnancies without medical assistance | 1,662 | 2,939,047 | 0.06 | 1 | 1 | Ref. | 598 | 1,491,607 | 0.04 | 1 | 1 | Ref. |
|  | ART | 30 | 44,116 | 0.07 | 1.20 | 1.09 | 0.75 to 1.58 | 9 | 52,041 | 0.02 | 0.43 | 0.37 | 0.19 to 0.73 |
|  | *Fresh-ET* | *27* | *39,049* | *0.07* | *1.22* | *1.10* | *0.74 to 1.61* | *6* | *39,109* | *0.02* | *0.38* | *0.33* | *0.14 to 0.74* |
|  | *Frozen-ET* | *3* | *5,067* | *0.06* | *1.04* | *1.05* | *0.33 to 3.28* | *3* | *12,932* | *0.02* | *0.58* | *0.52* | *0.17 to 1.62* |
| **32-36 weeks** | Pregnancies without medical assistance | 2,612 | 2,923,143 | 0.09 | 1 | 1 | Ref. | 986 | 1,484,735 | 0.07 | 1 | 1 | Ref. |
|  | ART | 45 | 43,613 | 0.10 | 1.16 | 1.08 | 0.80 to 1.45 | 39 | 51,585 | 0.08 | 1.14 | 1.14 | 0.82 to 1.58 |
|  | *Fresh-ET* | *43* | *38,590* | *0.11* | *1.25* | *1.15* | *0.85 to 1.57* | *31* | *38,744* | *0.08* | *1.21* | *1.20* | *0.83 to 1.73* |
|  | *Frozen-ET* | *<10* | *5,023* | *-* | *0.45* | *0.44* | *0.11 to 1.75* | *<10* | *12,841* | *-* | *0.94* | *0.94* | *0.47 to 1.90* |
| **37-41 weeks** | Pregnancies without medical assistance | 4,498 | 2,793,151 | 0.16 | 1 | 1 | Ref. | 1,918 | 1,424,620 | 0.13 | 1 | 1 | Ref. |
|  | ART | 86 | 40,615 | 0.21 | 1.32 | 1.02 | 0.82 to 1.27 | 65 | 48,445 | 0.13 | 1.00 | 0.85 | 0.66 to 1.10 |
|  | *Fresh-ET* | *70* | *35,891* | *0.19* | *1.21* | *0.93* | *0.73 to 1.18* | *51* | *36,287* | *0.14* | *1.04* | *0.89* | *0.67 to 1.18* |
|  | *Frozen-ET* | *16* | *4,724* | *0.34* | *2.11* | *1.73* | *1.05 to 2.84* | *14* | *12,158* | *0.12* | *0.85* | *0.74* | *0.43 to 1.25* |
| **42-44 weeks** | Pregnancies without medical assistance | 466 | 248,428 | 0.19 | 1 | 1 | Ref. | 94 | 86,131 | 0.11 | 1 | 1 | Ref. |
|  | ART | 8 | 3,139 | 0.25 | 1.36 | 0.98 | 0.44 to 2.21 | *3* | 2,606 | 0.12 | 1.05 | 0.88 | 0.27 to 2.84 |
|  | *Fresh-ET* | *<8* | *2,709* | *-* | *1.38* | *1.00* | *0.42 to 2.36* | *<8* | *1,704* | *-* | *0.54* | *0.44* | *0.06 to 3.20* |
|  | *Frozen-ET* | *<3* | *430* | *-* | *-* | *-* | *-* | *<3* | *902* | *-* | *-* | *-* | *-* |
| Abbreviations: CI - confidence interval, OR - odds ratio, Ref. - reference, fresh-ET - fresh embryo transfer, frozen-ET - frozen embryo transfer.  ^1^ Unadjusted. ^2^ Adjusted for maternal age, parity, country, year of birth.  ^3^ Observations from Denmark and Sweden were restricted to deliveries before April 2004 and July 2008, respectively, due to changes in definition of stillbirths (i.e. fetal deaths in week 22-27 were not reported to the Medical Births Registries before these time points). | | | | | | | | | | | | | |

**SUPPLEMENTAL MATERIAL**

**Sensitivity analyses of neonatal mortality using fetuses at risk as the denominator**

Studies of prenatal or pre-pregnancy risk factors for postnatal outcomes are prone to selection bias from conditioning on live birth.^1^ Exposures that increases the risk of miscarriage, pregnancy termination, or stillbirth will reduce the number of exposed in the live born population. ^1^ A time to event analysis of all pregnancies at risk of subsequent neonatal loss (i.e., fetuses assumed to be alive at the gestational age when the neonate who died was born), has been proposed as an alternative approach to the “denominator problem” in perinatal epidemiology ^2^. To investigate how selection through stillbirth influenced the results for neonatal death, we repeated the main analyses of neonatal death in the analysis sample that included stillbirths (sample A in Figure 1). Associations were estimated using Cox regression with gestational age as the time scale, where pregnancies ending before 45 completed weeks were followed from 22 weeks to delivery. Pregnancies ending with stillbirths, and live births with survival beyond the neonatal period, were censored at the time of delivery. This approach corresponds to the fetuses at risk approach.^2^ For comparison, we also repeated our main analysis (using multilevel logistic regression) in the live born singletons of sample A. The results are presented in Supplemental Table 6 and show that the two approaches give very similar association, which might also be expected when considering that we found no clear differences in overall risk of stillbirth between the conception method.

For the gestational-age-specific analyses of neonatal death, we also estimated rates of neonatal death per gestational week using the fetuses at risk approach in sample A, compared with the live birth approach (Supplemental Figure 2). Here, it should be noted that the fetuses at risk approach depends on the live birth rate at each gestational age, and therefore reflects the incidence of live births with a future neonatal death, rather than the incidence of neonatal death (3). In the live born population, gestational-age-specific neonatal mortality was similar for natural and ART conception, whereas in the fetuses at risk population, ART-conception was associated with a higher neonatal mortality in the second, but not in the third trimester. For prenatal exposures such as ART conception, which also increases the risk of preterm birth, none of the approaches can be used to obtain the direct effect on neonatal mortality (2). However, the predictive (prognostic) interpretation of the live birth denominator is still valid (2) and valuable for couples who experience a preterm birth after ART conception.

Our study population was limited to births of at least 22 completed weeks of gestation, and we could therefore not investigate whether pregnancy loss prior to this time point differed according to conception method.

| **Supplemental Table 6. Risk of neonatal death (0-27 days) according to conception method. Comparison of associations using ongoing pregnancies vs live births as the denominator** | | | |
| --- | --- | --- | --- |
| **Denominator / Model** | **Conception method** | **Hazard or odds ratio (95% CI), unadjusted ^1^** | **Hazard or odds ratio (95% CI), adjusted ^2^** |
|  |  |  |  |
| Fetuses at risk / Cox regression | Conceptions without medical assistance | 1 (Ref.) | 1 (Ref.) |
|  | Fresh-ET | 1.50 (1.30 to 1.74) | 1.63 (1.40 to 1.90) |
|  | Frozen-ET | 1.24 (0.90 to 1.71) | 1.57 (1.14 to 2.18) |
|  |  |  |  |
| Live births / Multilevel logistic regression | Conceptions without medical assistance | 1 (Ref.) | 1 (Ref.) |
|  | Fresh-ET | 1.46 (1.26 to 1.70) | 1.53 (1.31 to 1.78) |
|  | Frozen-ET | 1.31 (0.94 to 1.81) | 1.61 (1.16 to 2.23) |
| Abbreviations: CI - confidence interval, Ref. - reference, fresh-ET - fresh embryo transfer, frozen-ET - frozen embryo transfer.  ^1^ Unadjusted. ^2^ Adjusted for maternal age, parity, country, year of birth. | | | |

1. Snowden JM, Bovbjerg ML, Dissanayake M, Basso O. The curse of the perinatal epidemiologist: inferring causation amidst selection. Curr Epidemiol Rep 2018;5:379-87.

2. Harmon QE, Basso O, Weinberg CR, Wilcox AJ. Two denominators for one numerator: the example of neonatal mortality. European journal of epidemiology 2018;33:523-30.
