# Supplementary material for: Risk of stillbirth and neonatal death in singletons born after fresh and frozen embryo transfer. Cohort study from the Committee of Nordic Assisted Reproduction Technology and Safety
Source: Fertil Steril. Author manuscript; Available in PMC 2023 Nov 20. (PMC7615319; doi:10.1016/j.fertnstert.2022.10.020)
Supplement: Supplementary Figures [file EMS189986-supplement-Supplementary_Figures.pdf]

Author Accepted Manuscript version of the paper by Westvik-Johari et al in Fertil Steril, Vol 119 2023, DOI 10.1016/j.fertnstert.2022.10.020

Supplemental figure 1. Risk of stillbirth according to conception method and gestational age at birth. Upper panel: Unadjusted rates of stillbirth (per 1,000 fetal weeks), smoothed using a kernel function of moving averages. Interaction between conception method and gestational age was tested in Cox regression as a test of Schoenfeld residuals (proportional hazards test): p=0.0018 in unadjusted model and p<0.0001 in model with adjustment for parity, maternal age, offspring year of birth and country. Lower panel: Unadjusted cumulative risk of stillbirth as a percentage of all births (Kaplan-Meier curves). Log rank test p<0.0001.

CI: confidence interval, Fresh-ET: fresh embryo transfer, Frozen-ET: frozen embryo transfer.

Supplemental figure 2. Neonatal mortality according to conception method and gestational age. Upper panel: Postestimation from logistic models with adjustment for parity, maternal age, offspring year of birth and country. Likelihood ratio test of model with and without interaction term between conception method and gestational age: p=0.29. Lower panel: Unadjusted estimates using two different denominators: live births or fetuses at risk.

ART: Assisted reproductive technology, CI: confidence interval.

Distributed under the terms of the Creative Commons Attribution License (CC BY 4.0)

Figure(s)

Author Accepted Manuscript version of the paper by Westvik-Johari et al in Fertil Steril, Vol 119 2023, DOI 10.1016/j.fertnstert.2022.10.020

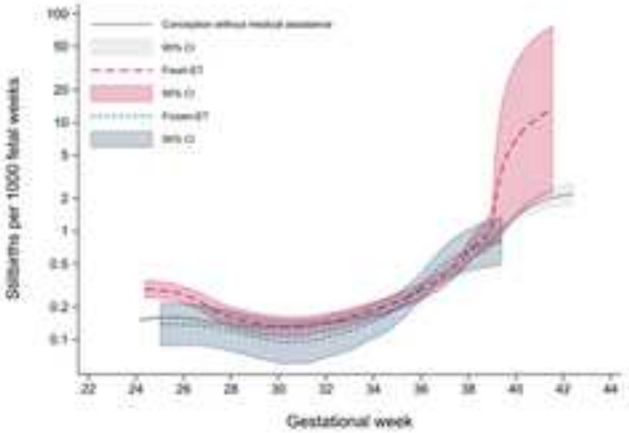

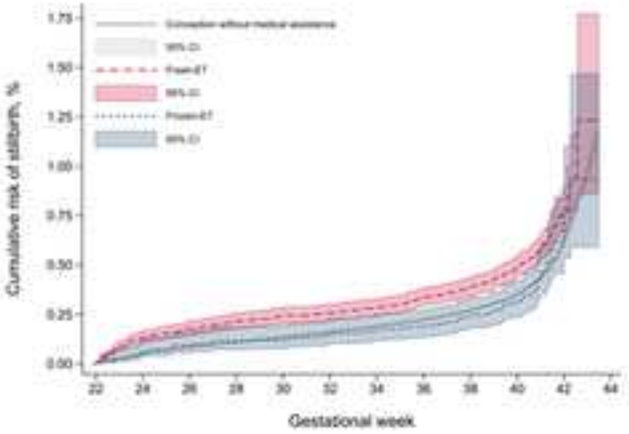

Distributed under the terms of the Creative Commons Attribution License (CC BY 4.0)

Figure(s)

Author Accepted Manuscript version of the paper by Westvik-Johari et al in Fertil Steril, Vol 119 2023, DOI 10.1016/j.fertnstert.2022.10.020

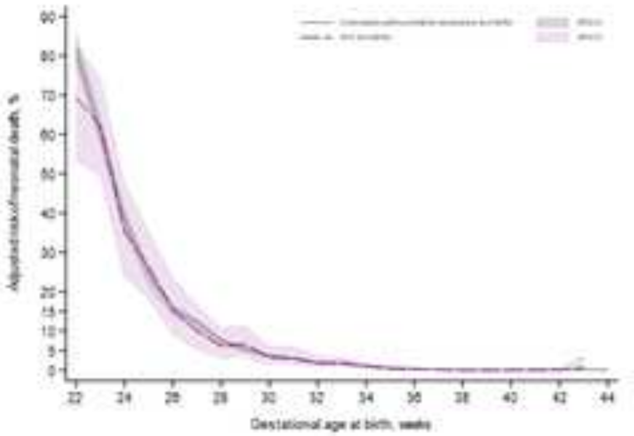

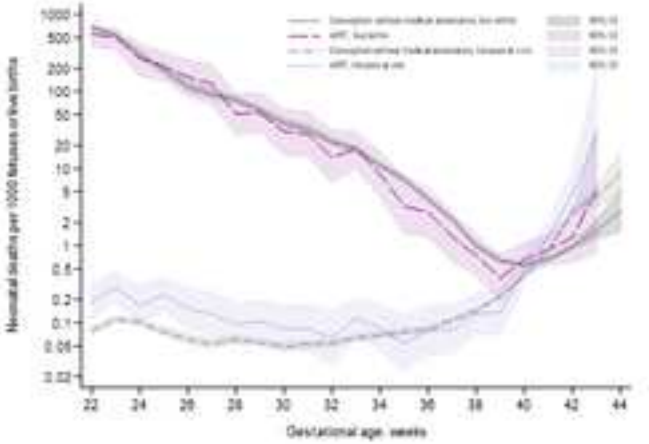

Distributed under the terms of the Creative Commons Attribution License (CC BY 4.0)
